# Response to the Letter to the Editor regarding “Lateral versus posterior quadratus lumborum block in children undergoing open orchiopexy: a double-blind randomized clinical trial”

**DOI:** 10.1016/j.bjane.2025.844718

**Published:** 2025-12-06

**Authors:** Ozgecan P. Zanbak Mutlu, Pinar Kendigelen, Ayse C. Tutuncu

**Affiliations:** Istanbul University-Cerrahpasa, Cerrahpasa Faculty of Medicine, Department of Anesthesiology and Reanimation, Istanbul, Turkey

Dear Editor,

We sincerely thank Dr. Aphale and colleagues for their interest in our study and for their appreciative comments that underscore the value of our study entitled *“*Lateral versus posterior quadratus lumborum block in children undergoing open orchiopexy: a double-blind randomized clinical trial”.^1^ Their thoughtful remarks provide an opportunity to clarify the scope and objectives of our research and to emphasize several methodological considerations.^2^

As correctly noted,^2^ current systematic reviews and meta-analyses have indicated that Quadratus Lumborum Block (QLB) is an effective and reliable postoperative analgesic technique for children undergoing lower abdominal surgeries, providing longer and superior pain relief compared with caudal anesthesia.^3^ Our study, however, was not designed to compare QLB with caudal block or systemic analgesia. The primary aim was to compare the analgesic efficacy of two QLB approaches applied from different anatomical sites ‒ lateral and posterior ‒ in a specific pediatric surgical procedure, open orchiopexy.^1^ Therefore, including a control group such as a caudal or systemic analgesic was beyond the scope of our study. Nevertheless, we agree that future randomized controlled studies comparing different QLB approaches with caudal and systemic analgesic techniques in different types of surgeries are warranted to identify the optimal approach and further strengthen clinical practice.

Regarding the comment on the absence of dermatomal mapping,^2^ we acknowledge that standardized sensory evaluation could provide additional insight into the spread of the local anesthetic. However, as clearly stated in our manuscript, sensory testing was not feasible in our study population due to the use of general anesthesia and the young age of participants. This limitation was explicitly addressed in the discussion section.^1^ In pediatric patients, particularly under general anesthesia, objective sensory assessment poses both practical and ethical challenges; therefore, the dermatomal distribution of local anesthetics cannot be precisely delineated in a clinical setting.

Additionally, we thoroughly discussed that both lateral and posterior QLB may not be sufficient for scrotal incisions because of the complex and variable innervation of this region. As noted in our manuscript, this observation was attributed to anatomical variations in local anesthetic spread and uncovered innervation zones. Nevertheless, there were no statistically significant differences between the two groups regarding the additional analgesic requirements, nor in the duration of analgesic-free intervals.^1^

We also noted the concern regarding parental pain scoring after discharge. As mentioned in our methods and limitations, pain assessment by caregivers is an inherent constraint of pediatric outpatient surgery research.^1^ To minimize the limitation parents were pre-instructed on the use of the Wong-Baker Pain Scale, and written guidance was provided before discharge. Similar approaches are widely used in the pediatric anesthesia literature for day-case surgeries, given the impracticality of inpatient follow-up for every patient.^4^ Hence, while subjective, parental assessment remains an accepted and pragmatic method for short-term postoperative evaluation in this setting.

We concur that several factors influence the choice of regional anesthesia technique in clinical practice, including the type of surgery, patient positioning, anesthesiologist experience, and anticipated spread of local anesthetic. Our findings demonstrated that lateral and posterior QLB techniques produced comparable analgesic efficacy for open orchiopexy.^1^ Thus, given the technical simplicity of the lateral approach, this method may be a reasonable alternative in clinical scenarios where both efficacy and ease of performance are relevant considerations.

Finally, to the best of our knowledge, our study was the first double-blind, prospective, randomized trial comparing lateral and posterior QLB in children. We fully agree that further research is warranted to evaluate the comparative analgesic effectiveness of different QLB approaches across various pediatric surgical procedures. Such studies should ideally incorporate imaging-based confirmation of block spread and consider long-term pain outcomes when ethically and logistically feasible. However, the assessment of long-term outcomes was beyond the scope of our hypothesis. We specifically designed our study to evaluate the first 24 postoperative hours, which represent the period when postoperative pain is most intense.^1^^,^^5^

In conclusion, we appreciate the insightful feedback from Dr. Aphale and colleagues.^2^ We hope our clarifications reinforce the intended focus of our study and contribute to ongoing efforts to optimize regional anesthesia strategies in children.

## Data availability statement

The datasets generated and/or analyzed during the current study are available from the corresponding author upon reasonable request.

## Authors’ contributions

Ozgecan P. Zanbak Mutlu made substantial contributions to the conception, interpretation, and drafting of the manuscript.

Pinar Kendigelen contributed to revision of the manuscript.

Ayse C. Tutuncu played a key role in the study's conception and critical revision of the manuscript.

## Funding

None.

## Conflicts of interest

The authors declare no conflicts of interest.
